# Fisetin as an adjuvant treatment in prostate cancer patients receiving androgen-deprivation therapy

**DOI:** 10.2144/fsoa-2022-0002

**Published:** 2022-02-11

**Authors:** Giuseppe Di Lorenzo, Luca Scafuri, Ferdinando Costabile, Liuba Pepe, Anna Scognamiglio, Felice Crocetto, Germano Guerra, Carlo Buonerba

**Affiliations:** 1 Oncology Unit, Hospital ‘Andrea Tortora’, ASL Salerno, Pagani, Italy; 2 Associazione O.R.A., Somma Vesuviana, Naples, Italy; 3 Department of Medicine & Health Science, University of Molise, Campobasso, Italy; 4 Department of Neurosciences, Reproductive Sciences & Odontostomatology, Federico II University, Naples, Italy

**Keywords:** androgen deprivation therapy, fisetin, prostate cancer

Prostate cancer represents the second most frequently occurring malignancy in males, with about 1.4 million men estimated to have been diagnosed with prostate cancer in 2020 worldwide [1]. In spite of significant advances in systemic treatment of advanced prostate cancer over the last decade [2–4], androgen deprivation therapy (ADT) still represents the backbone of systemic treatment of advanced prostate cancer, and is usually administered for years. Long-term ADT is associated with several and diverse adverse events, which include dysfunctions in glucose and lipid metabolism, osteoporosis and increased cardiovascular risk [5]. Furthermore, a mounting body of evidence indicates that ADT may be associated with deterioration of cognitive functions. One of the largest studies conducted on the topic analyzed a dataset obtained from the Cancer of the Prostate Strategic Urologic Research Endeavor (CaPSURE) registry, and included 13,570 men aged ≥50 years of whom 317 (2.3%) were diagnosed with dementia. Of note, multivariate analysis showed that ADT was significantly associated with dementia (HR: 2.02; 95% CI: 1.40–2.91; p < 0.01). This association was confirmed in a subset of 8506 men who were matched using propensity score according to whether they had or had not received ADT (HR: 1.59; 95% CI: 1.03–2.44; p = 0.04). Finally, this study did not reveal any association between dementia and primary treatment type in the subgroup of 8489 men who were not treated with ADT [6].

Fisetin is a naturally occurring flavonol, and is currently marketed and available worldwide mostly as a supplement containing extracts from *Cotinus coggygria* [7]. Besides having antioxidant, anti-inflammatory and antiproliferative activity, fisetin has recently gained attention in the medical community, mostly because of its potential effect against senescent cells, which are resistant to apoptosis and may be involved both in physiologic aging and in multiple pathologic conditions [8]. Long-term oral supplementation with fisetin is expected to be associated with few adverse events at the daily dose of 200 mg [9] and may be particularly advantageous for patients with advanced prostate cancer receiving ADT for several reasons. First, fisetin may help reduce the increased cardiovascular risk via multiple biological mechanisms. In a murine model of cardiac ischemia-reperfusion injury, oral fisetin (20 mg/kg) administered for 28 days yielded a significant upregulation of PPAR-γ expression in the heart, which was associated with reduced levels of inflammation and cardiac injury markers, decreased oxidative stress, inhibition of apoptosis and reduced infarction size [10]. Consistent results have been obtained in an *in vitro* study showing that fisetin was associated with inhibition of apoptosis and decreased reactive oxygen species generation in a culture of rat cardiomyocytes [11]. Also, fisetin yielded a range of favorable metabolic effects in a high-fat diet mouse model, which included reduction of body weight, as well as insulin and fasting blood glucose levels [12]. These favorable effects may be mediated by decreased gluconeogenic and glycogenolytic activity in the liver, as shown in other experimental mouse models reporting that fisetin administration was associated with decreased expression of phosphoenolpyruvate carboxykinase and glucose-6-phosphatase genes and inhibition of glucose 6-phosphatase activity [13,14]. Importantly, it must be considered that fisetin reduced cognitive deficits in rapidly aging senescence-accelerated prone 8 mice and was capable of restoring several markers of impaired synaptic function, stress and inflammation [15]. Fisetin may also prevent accumulation of amyloid beta [16]. Importantly, there is proof that fisetin exerts direct antineoplastic activity against prostate cancer cells, alone [8] or in combination with cabazitaxel [17], a drug that represents the backbone of chemotherapy treatment of advanced prostate cancer [18,19]. Finally, fisetin may also contribute to the prevention of infection with SARS-CoV-2 [20–22].

In conclusion, fisetin may be tested as part of a nutritional intervention in men receiving long-term ADT to protect them against some of the most clinically relevant associated adverse events. Fisetin may even enhance antineoplastic activity of ADT or other concomitant agents. Optimal dosing remains to be determined. Based on ongoing clinical trials involving fisetin, it is likely that a daily dose in the range of 500–1000 mg can be selected for initial clinical testing in this setting.
